# Nasopharyngeal Carcinoma: The Role of the EGFR in Epstein–Barr Virus Infection

**DOI:** 10.3390/pathogens10091113

**Published:** 2021-08-31

**Authors:** Xintong Peng, Yanling Zhou, Yongguang Tao, Shuang Liu

**Affiliations:** 1 Key Laboratory of Carcinogenesis and Cancer Invasion, Ministry of Education, Department of Pathology, Xiangya Hospital, Central South University, Changsha 410078, China; xtPeng@csu.edu.cn; 2 Key Laboratory of Carcinogenesis of the Ministry of Health, Cancer Research Institute, School of Basic Medicine, Central South University, Changsha 410078, China; 3 Department of Oncology, Institute of Medical Sciences, National Clinical Research Center for Geriatric Disorders, Xiangya Hospital, Central South University, Changsha 410078, China; yanlingzhou@csu.edu.cn; 4 Department of Thoracic Surgery, Hunan Key Laboratory of Tumor Models and Individualized Medicine, Second Xiangya Hospital, Central South University, Changsha 410078, China

**Keywords:** Epstein–Barr virus, EGFR, LMP1, nasopharyngeal carcinoma, EGFR-targeted agents

## Abstract

Epstein–Barr virus (EBV), a type 4 γ herpes virus, is recognized as a causative agent in nasopharyngeal carcinoma (NPC). Incidence of EBV-positive NPC have grown in recent decades along with worse outcomes compared with their EBV-negative counterparts. Latent membrane protein 1 (LMP1), encoded by EBV, induces NPC progression. The epidermal growth factor receptor (EGFR), a member of the ErbB family of receptor tyrosine kinases (RTK), is a driver of tumorigenesis, including for NPC. Little data exist on the relationship between EGFR and EBV-induced NPC. In our initial review, we found that LMP1 promoted the expression of EGFR in NPC in two main ways: the NF-κB pathway and STAT3 activation. On the other hand, EGFR also enhances EBV infection in NPC cells. Moreover, activation of EGFR signalling affects NPC cell proliferation, cell cycle progression, angiogenesis, invasion, and metastasis. Since EGFR promotes tumorigenesis and progression by downstream signalling pathways, causing poor outcomes in NPC patients, EGFR-targeted drugs could be considered a newly developed anti-tumor drug. Here, we summarize the major studies on EBV, EGFR, and LMP1-regulatory EGFR expression and nucleus location in NPC and discuss the clinical efficacy of EGFR-targeted agents in locally advanced NPC (LA NPC) and recurrent or metastatic NPC (R/M NPC) patients.

## 1. Epstein–Barr Virus (EBV)

### 1.1. Etiology of EBV

EBV was first described in 1958 by Dr. Denis Burkitt, who identified clusters of mandibular sarcomas in African children aged 2 to 14 [1]. Then, he met virologist Anthony Epstein by chance to find out if there was a viral cause of this malignant lymphoma. After years of research, Dr. Epstein provided essential insights into the association between EBV and human disease [1]. The EBV is a type 4 γ herpes virus, comprising lipoprotein capsules and 162 shell particles [2]. The genome comprises 190 kb double-stranded DNA, encoding about 100 open-reading frames [1,3]. Free circular EBV DNA usually exists in the cytoplasm of lymphocytes and can eventually be integrated into lymphocyte chromatin [2]. The latent EBV protein promotes the proliferation of host cells and boosts DNA replication of EBV in host cells by two means: infected B-cell proliferation or lytic virion production [4]. The existence of EBV culminates in disease.

### 1.2. EBV Causes Disease by Infecting B Cells and Epithelial Cells

It has been reported that EBV can infect both epithelial cells and B cells in vivo and shuttle back and forth between them [5]. This phenomenon is conducive to the multiplication and survival of EBV; however, the specific infection mechanisms of B lymphocytes and epithelial cells are quite different [6]. In B cells, EBV uses its envelope protein gp350 to bind to surface CD21 receptors, and gp42 to bind to HLA-II proteins to transform B cells into immortalized B lymphoblastic cell lines (LCLs). These establish a latent infection status [4,7], which depends on a group of potential genes, including six types of EBV nuclear antigens––EBNA 1, 2, 3A, 3B, and 3C and EBNA lead protein (EBNA-LP)––latent membrane proteins LMP1 and LMP2 (including LMP2A and LMP2B), EBV-encoded small RNAs (EBER1 and EBER2), and microRNAs (miRNAs) [1,2].

On the other hand, EBV infection of epithelial cells is much more complicated. The study showed that EBV-positive epithelial cells are bloodier than EBV-negative epithelial cells [8]. A recent study suggested that EphA2 and Neuropilin-1(NRP1) allow the EBV to enter epithelial cells [9,10], as does EBV gHgL, which interacts with the integrin complex αvβ6 and αvβ8 to trigger the fusion of the EBV envelope protein with the cell membrane [11].

The particles released by EBV-infected epithelial cells are rich in gp42, whereas the particles released by EBV in B cells are deficient. Interestingly, gp42 can affect the formation of the gHgL complex, thus preventing the EBV from entering epithelial cells. This bidirectional regulation promotes EBV shuttling between B cells and epithelial cells. It is worth mentioning that EBV infection cannot immortalize epithelial cells and requires direct epithelial-to-epithelial contact.

According to the diverse transcription of latent genes, there are four latencies. When EBERs are solely transcribed, it is type 0 latency, mainly occurring in dormant memory B cells [4]; when EBERS, EBNA1, and BARTS are simultaneously transcribed, it is type I latency, mainly in Burkitt’s lymphoma [12]; when EBER, EBNA1, LMPS, and BARTS are concurrently expressed, it is type II latency, commonly seen in Hodgkin’s lymphoma, gastric cancer, and NPC; and when EBERS, EBNA1, EBNA-LP, EBNA2, EBNA3A, EBNA3B, EBNA3C, and LMPs are synchronously transcribed, it is type III latency, primarily appearing in the condition of immunodeficiency [13]. Different transcriptional statuses provide an essential reference for the occurrence of various diseases.

### 1.3. EBV Infection can Promote the Progression of Nasopharyngeal Carcinoma

EBV infection is common worldwide, with a prevalence of 80–95%, depending on the geographical area [14]; however, people are predominantly asymptomatic [14]. Both the means of infection and the host factors may affect the outcomes of EBV infection, which mainly infects the host through lymphocytes and epithelial cells [2,3,15]. For example, infection in adolescents can lead to infectious mononucleosis, with manifestations of fever, sore throat, lymphadenectasis, and splenomegaly [16]. Burkitt’s lymphoma (BL) and classical Hodgkin’s lymphoma (HL) are related to the EBV infection of lymphocytes, whereas nasopharyngeal and gastric cancers are associated with the infection of epithelial cells [2]. In particular, NPC is a frequently reported tumor in the southeastern provinces of China, and almost 98% of NPCs are closely related to EBV infection [5].

A study of molecular mechanisms proved that the invasion and migration ability was significantly up-regulated in EBV-positive NPC cells. At the same time, the tumor formation ability of EBV-positive NPC cells was significantly higher than EBV-negative NPC cells in nude mice. In short, the malignant degree of nasopharyngeal carcinoma cells was increased in the presence of EBV in both in vivo and in vitro experiments [17]. Meanwhile, substantial scientific studies have shown that the level of free EBV DNA in the plasma of NPC patients is highly correlated with the prognosis. Patients with low EBV DNA content have shown a better prognosis, which has excellent clinical guiding significance. EBV has been used as a clinical marker in the clinical diagnosis of NPC [18].

### 1.4. LMP1 Protein Encoded by EBV Is Involved in the Progression of Nasopharyngeal Carcinoma

LMP1 is a membrane protein encoded by EBV, consisting of a short amino acid N-terminal, six hydrophobic alpha-helical transmembrane regions, and a large 200-amino acid cytoplasmic C-terminal tail [19]. LMP1 contains several domains, and the C-terminal which contains three functional domains, CTAR1, CTAR2, and CTAR3. Only CTAR1 uniquely induces several cellular genes, including the epidermal growth factor receptor (EGFR), TRAF1, ICAM1, and EBI3 [20]. LMP1 contributes to the development and progression of NPC by many mechanisms, including the regulation of the expression and phosphorylation of the transcription factor p53 [21], the EGFR and the STAT3 activation allowance [22], as well as necroptosis inhibition through the RIP3 promoter hypermethylation [23]. 

## 2. Epidermal Growth Factor Receptor (EGFR)

### 2.1. Biological Function of EGFR

EGFR (also known as ErbB1 or HER1) is a 170kDa single transmembrane glycoprotein of the receptor tyrosine kinase family (RTKs), and a member of the ErbB family (EGFR, HER2, HER3, and HER4), and is essential for epithelial cell biology [24,25]. ErbB family receptors contain an extracellular N-terminus extracellular ligand-binding domain, a hydrophobic transmembrane domain, and a conserved cytoplasmic C-terminus tyrosine kinase domain [26]. Growth factors could activate ErbB by autocrine or paracrine signalling [27]. Because the ligand binds to the extracellular domain of ErbB, it induces receptor homodimer or heterodimer formation, which phosphorylates tyrosine residues in the cytoplasmic tail, which then activates the intracellular tyrosine kinase domain [28]. Indeed, the autophosphorylation of ErbB family members is a critical step in downstream signal transduction that affects cell survival, proliferation, angiogenesis, migration, inflammatory responses, and oncogene expression [29,30].

**Figure 1:**
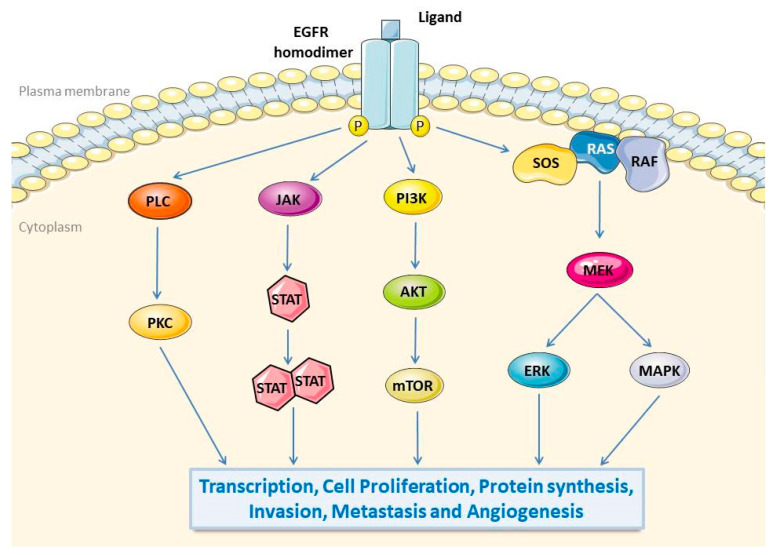
EGFR signalling pathways. The binding of ligands in the extracellular domain of EGFR induces the formation of dimers and then phosphorylate tyrosine residues in the intracellular domain, leading to activated downstream signal transduction. EGFR intracellular signalling cascades include: (1) PKC pathway, (2) JAK/STAT pathway, (3) PI3K/Akt/mTOR pathway, (4) Ras/Raf/MEK/MAPK/ERK pathway. Subsequent EGFR downstream pathways affect gene transcription, proliferation, protein synthesis, invasion, metastasis, and angiogenesis. EGFR, epidermal growth factor receptor; PKC, protein kinase C; JAK, Janus kinase; STAT, signal transduction and transcriptional activator protein; PI3K, phosphoinositide-3 kinase; Akt, v-akt murine thymoma viral oncogene homolog 1; mTOR, mammalian target of rapamycin; Ras, retroviral associated DNA sequence; Raf, v-Raf 1 murine leukemia viral oncogene homologue 1; MAPK, mitogen-activated protein kinase; ERK, extracellular signal-regulated kinase.

Currently, the known downstream signalling cascades of EGFR follow five pathways: (1) Ras/Raf/MEK/mitogen-activated protein kinase (MAPK)/ERK, (2) phosphatidylinositol 3-kinase (PI3K)/Akt/mTOR, (3) protein kinase C (PKC), (4) Src, and (5) Jak/STAT (Figure 1) [31,32,33]. In addition, the canonical receptor-dependent EGFR signalling pathway and the ligand-independent and tyrosine kinase-independent EGFR novel mechanisms were recently discovered, revealing that EGFR also plays a regulatory role in autophagy and metabolism [34]. In short, both EGFR and the EGFR downstream signalling pathways are necessary for cell fate and influence tumorigenesis.

### 2.2. EGFR Promotes Tumorigenesis and Progression

EGFR alterations are common in many malignant tumors such as lung, breast, stomach, and colorectal cancer and glioblastoma [32]. The dominating triggers of EGFR activation in tumor tissues are EGFR gene amplification and point mutations. Transcriptional upregulation or ligand overproduction caused by autocrine/paracrine secretion has also been shown [35]. Overexpression of the EGFR can lead to a poor prognosis, drug tolerance, tumor metastasis, and a low survival rate [36,37,38]. As mentioned above, the ligand stimulates the homologous or heterologous dimerization of the receptor. It ultimately leads to the activation of the kinase, which contributes to enhanced EGFR signalling and promotes tumor development [37,39]. Because the EGFR is frequently mutated and overexpressed in tumors, it is also a promising therapeutic target for a host of tumors in clinical trials [35]. In brief, EGFR signalling is correlated with tumor proliferation, invasion, and metastasis [40].

The dimerization of ErbB allows intracellular signal transduction and tumorigenesis. The most effective of all the ErbB dimers is the ErbB-2/ErbB-3 heterodimer complex. ErbB-2/ErbB-3 dimers promote cell proliferation and transformation in vitro. They are further involved in the pathogenesis of lung cancer and breast cancer [41]. Considering the components of the heterodimer ErbB-2/ErbB-3, ErbB-2 is an excellent partner for forming a heterodimer because it is a ligand-less co-receptor; ErBb-3, a kinase-activated impaired receptor, cannot be internalized and degraded in lysosomes to prolong signalling and promote cell transformation [42].

Furthermore, ErbB-2/ErbB-3 has more vital signal transduction ability because it transmits proliferation signals not only through the Ras/Raf/MAPK pathway but also the PI3K/Akt/mTOR pathway [39]. Since a heterodimer has a weaker binding strength compared to a homodimer, it is easy to dissociate in the endosome. In addition, it cannot recruit the E3 ligase Cbl to initiate endocytosis to degradation, so the heterodimer is enhanced, which makes it more tumorigenic than the homodimer [35]. Obviously, in tumor signal transduction, ErbB heterodimers, particularly ErbB-2/ErbB-3, are more effective than homodimers.

## 3. The Mechanism of LMP1-Mediated EGFR Expression and Nuclear Translocation

The EBV can encode a protein mass to play a role in tumorigenesis. Existing studies have shown that only LMP1 takes part in the activation of the EGFR in NPC. Initially, Miller et al. found that EGFR expression was significantly increased when LMP1 was stably expressed in epithelial cells, which could be activated by the EGF ligands [43]. Further studies suggested that the expressions of LMP1 further led to the growth and differentiation of epithelial cells. However, regardless of its levels, LMP1 could not affect the EGFR level in B cells [43]. To clarify the relationship between LMP1 and EGFR in exosomes, researchers separated the exosomes of C666 and C666–LMP1 cells. They found that the EGFR content increased significantly after the overexpression of LMP1 [44]. More interestingly, studies found that LMP2A can inhibit the expression of LMP1 and the activation of the NF-κB pathway [45].

It is known that there are three domains in the C-terminal of LMP1, but which are involved in this process? Subsequent studies found that the CTAR1 domain at the carboxyl end of LMP1 can drive the up-regulation of the EGFR, while the CTAR2 domain cannot [46]. In-depth research found that the EGFR levels of CTAR1-solely expressed C33A cells are significantly higher than LMP1-expressed C33A cells, indicating that the presence of CTAR2 may impede the ability of CTAR1 [46]. We increased the expression of LMP1 by Tet-on in HNE2 cells then measured the EGFR expression in the nucleus by a double immunofluorescent stain using a fluorescein isothiocyanate (FITC)-conjugated anti-EGFR antibody and enhanced green fluorescent protein. The study further found that LMP1 can promote EGFR expression and facilitate the nuclear localization signal (NLS)-mediated nuclear translocation of the EGFR independent of the enhanced green fluorescent protein [47,48]. 

**Figure 2:**
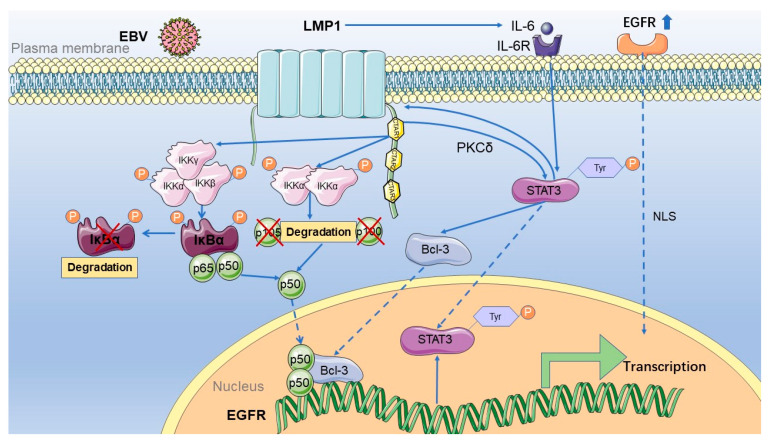
The mechanism of EGFR activation by LMP1 in nasopharyngeal carcinoma. LMP1 activates the EGFR mainly in two ways: first, the expression encoded by EBV activates the NF-κB classical and non-classical pathways, and then p50 can enter the nucleus. Meanwhile, LMP1 can regulate the phosphorylation of STAT3 tyrosine 705, which PKCδ mediates. It then activates the expression of Bcl-3 and promotes it to enter the nucleus. Indeed, p50 and Bcl-3 can form a trimer or dimer, binding to the EGFR promoter to activate its transcription. Activated STAT3 can then bind to the LMP1 promoter to regulate the expression of LMP1. At the same time, IL-6 downstream of the NF-κB pathway activated by LMP1 can further activate STAT3 after binding to its receptor. Moreover, STAT3 then binds to the LMP1 promoter to promote the expression of LMP1, forming a positive autoregulatory loop. STAT3 is not only regulated by LMP1 but also regulated by the activation of EGFR. IKKα, IkappaB kinase-alpha; IKKβ, IkappaB kinase-beta; IKKγ, IkappaB kinase-gamma; IκBα, IkappaBalpha; LMP1, latent membrane protein 1; EBV, Epstein–Barr virus; Bcl-3, B-cell lymphoma 3; STAT3, signal transducer and activator of transcription 3; EGFR, epidermal growth factor receptor; NLS, nuclear localization protein; CTAR, carboxyl-terminal activating regions; IL-6, interleukin 6; IL-6R, interleukin 6 receptor.

Studies have shown that LMP1 mediates the expression of the EGFR in nasopharyngeal carcinoma in several ways, including via the NF-κB pathway and STAT3 activation (Figure 2).

### 3.1. LMP1 Activates the EGFR through the NF-κB Pathway in NPC

NF-κB is a family of transcription factors regulating various biological processes, including inflammation, apoptosis, cell cycle, and cell migration [49]. The mammalian NF-κB transcription factor family consists of five members: p65 (RelA), c-Rel, RelB, p50, and p52, all containing a Rel homology domain [50]. These five transcription factors can form dimers with each other and bind to specific sites in DNA [51]. Among them, only p50/p65 physiologically exist in the cytoplasm due to the existence of IkBα. However, when some stimuli cause IkBα to degrade, p50/p65 dimers can enter the nucleus. This pattern is the classic NF-κB pathway [52], in addition to which there is a nuclear translocation regulation (non-classical) pathway where p50 and p52 are restricted in the cytoplasm by their precursor proteins p105 and p100, respectively. After p105 and p100 are cleaved, p50 and p52 can enter the nucleus [53,54].

LMP1 contains three domains, CTAR1, CTAR2, and CTAR3; however, only CTAR1 is involved in the induction of the EGFR promoter, while CTAR2 cannot mediate the EGFR promoter. What type of NF-κB is activated by LMP1? It has been reported that both of them can activate the NF-κB pathway: LMP1–CTAR2 activates the classical NF-κB signal, and LMP1–CTAR1 induces more complex NF-κB signalling, including classical and non-classical pathways [52,55]. The classical NF-κB pathway contains the IKKα, IKKβ, and IKKγ enzymes. By contrast, the non-classical NF-κB pathway contains the enzyme IKKα activated by NIK. Studies have shown that the knock-out of IKKα, IKKβ, and IKKγ does not affect LMP1-mediated EGFR up-regulation. However, the EGFR could be down-regulated after the knock-out of NIK, proving that the EGFR expression induced by LMP1 is not necessarily dependent on the classical and alternative proteasome-dependent NF-κB pathway [52].

Specifically, the two domains of the C-terminal of LMP1, CTAR1, and CTAR2, can activate different NF-κB transcription factor dimers, respectively. CTAR1 activates the p50/p50, the p50/p52, and the p52/p65 dimers, but CTAR2 only activates the p52/p65 dimer [56], which initially inhibits transcription but can acquire the transcriptional activity after combining with B-Cell Chronic Lymphocytic Leukaemia/Lymphoma-3 (Bcl-3) [57]. The level of p50 in the nucleus is significantly up-regulated by LMP1 and LMP1–CTAR1, which verifies that LMP–CTAR1 could induce the nuclear translocation of p50 [58], and that the p50 homodimer could then bind to the NF-κB binding site in the EGFR promoter [59]. The p50/Bcl-3 complex can bind to the promoter region of the EGFR. In short, LMP1 activates the NF-κB pathway to form p50 dimers, thus activating Bcl-3, and then forming the p50/Bcl-3 complex that binds to the EGFR promoter region to up-regulate EGFR expression [43,60]. In the analysis of NPC clinical samples, the expression level of p50 in the nucleus was significantly higher than in low LMP1 level samples, which supports the previous hypothesis [43]. Detections of C33A cells and NPC tissues by chromatin immunoprecipitation (ChIP) verified that p50 molecules can bind to the promoter region of the EGFR, making the molecular mechanism clear [51]. Interestingly, after knocking down p105 and p50, the expression level of Bcl-3 was significantly increased. The formation of Bcl-3 and p50 was increased, suggesting that p105 or p50 may have a negative effect on Bcl-3 [58]. In conclusion, LMP1 activates EGFR transcription by the p50/ Bcl-3 complex. Further studies are required to determine the links between other signal-transducing complexes and their contributions to NPC.

### 3.2. LMP1 Activates EGFR through STAT3 in NPC

The activation of STAT3 (signal transcription and signal activator 3) is related to a variety of epithelial and lymphatic system malignancies, such as breast cancer [61], multiple myeloma [62], and NPC [63]. Its transcriptional activity is regulated by phosphorylation. In particular, phosphorylation of STAT3 tyrosine 705 can induce Bcl-3 dimerization, while phosphorylation of STAT3 serine 727 can affect DNA binding and transcriptional activity [46]. However, inhibiting PKCδ by Rottlerin (PKCδ inhibitor) decreased LMP1–CTAR1-induced serine phosphorylation but not tyrosine phosphorylation, even though the EBV-encoded LMP1, can activate STAT3, which requires PKCδ [64]. The results of the two studies contradicted each other. Further research is needed to clarify this dilemma.

LMP1–CTAR1 can further upregulate the EGFR after STAT3 activation because CTAR1 promotes the tyrosine phosphorylation and activation of STAT3, which further induces the expression of Bcl-3 and the nuclear translocation of Bcl-3 and p50. Bcl-3 binds with p50 as a transcriptionally active complex to activate the EGFR expression [46]. Interestingly, activated STAT3 could bind to the L1–TR LMP1 promoter in the nucleus. Therefore, LMP1 expression was upregulated through the JAK–STAT pathway, and STATs predisposed the cell to EBV-driven tumorigenesis [65]. 

Meanwhile, LMP1 increases IL-6 synthesis by the NF-κB pathway. IL-6 further mediates tyrosine phosphorylation of STAT3 after binding to its receptor, after which the activated STAT3 binds to the LMP1 promoter to promote the expression of LMP1, forming a positive autoregulatory loop [66]. Moreover, STAT3 is not only regulated by LMP1 but also by the activation of the EGFR [67]. ChIP was used to detect NPC clinical samples and C33A cells and verified that Bcl-3 molecules could bind to the EGFR promoter region [58]. In short, the activation of STAT3 and Bcl-3 is critical to the transcription of the EGFR. Crosstalk between STAT3 activation and NF-κB was found in the presence of Bcl-3, but the mechanism is still not entirely clear.

### 3.3. Others

The ChIP analysis of CTAR1-expressing C33 cells indicated that both PIK3R1 and PIK3R3 bound to Bcl3, but the pattern remains unknown [20]. There are still various mechanisms to be investigated that are associated with LMP1 and the EGFR.

## 4. The Role of EGFR Pathways in Nasopharyngeal Carcinoma

### 4.1. The Relationship between EGFR and Nasopharyngeal Carcinoma

EGFR mutation recurs in various tumors except for NPC, and EGFR overexpression is quite common in NPC [68]. The TCGA analysis revealed that patients with a high expression of EGFR mRNA had a poorer prognosis than those with a low expression [69]. In addition, the EGFR was co-expressed with LMP1 in most NPC tissues examined by immunostaining and in situ hybridization experiments [70,71]. Several studies also showed that EGFR expression correlated with the advanced tumor node metastatic stage, clinical stage, and distant metastatic state of NPC patients by analyzing a cohort of clinical samples [72]. Consequently, it is a potential prognostic biomarker for advanced-stage patients with a poor outcome [73]. 

### 4.2. EGFR Signalling Affects the Growth of Nasopharyngeal Carcinoma Cells

In NPC cells, the abnormal expression of EGFR regulates the cell cycle and tumor growth by related genes. For example, in LMP1-positive NPC tissues of elderly individuals, the expression of the EGFR was closely related to the high enrichment of p53 in the nucleus and the expression of Bcl-2, suggesting that the up-regulation of the EGFR or Bcl-2 was associated with a poor prognosis and resistance to chemotherapy-induced apoptosis [74]. As mentioned above, LMP1 promotes EGFR binding to the promoter region of cyclin E and cyclin D1, thus accelerating the G1/S phase transition of cells [22,47]. In addition, microRNAs (miRNAs) also influence the proliferation of NPC cells through the EGFR pathway. For instance, VPS33B (vacuolar protein sorting 33B) up-regulates miR-133A-3 to suppress cell growth and induce cycle arrest by the EGFR/PI3K/Akt/c-myc/p53 pathway. Interestingly, p53 induces the expression of miR-133a-3p- to form a feedback loop. Therefore, VPS33B may be a new molecule target for developing novel NPC therapeutic methods [75].

### 4.3. EGFR Promotes Invasion and Metastasis of Nasopharyngeal Carcinoma Cells

EGFR signalling can promote PKM2 nuclear translocation, which stimulates the transcription of FosL1, ANTXR2, CCND1, cyclin D1, and c-Myc genes. The expression of these genes promotes cell cycle progression and the Warburg effect, which enhances the invasion and metastasis ability of NPC cells [72,76]. Moreover, the highly conserved transcription factors Forkhead box Q1 (Foxq1), regulated by miR-124, could directly bind to the EGFR promoter and increase EGFR expression, thereby inducing vasculogenic mimicry via the EGFR signalling pathway to promote NPC metastasis [77,78]. Similarly, the overexpression of LACTB (serine beta-lactamase-like protein) promotes NPC cell motility in vitro and metastasis in vivo, depending on the activation of ErbB3/EGFR–ERK signalling, which is not conducive to the survival of NPC patients [79]. On the contrary, the overexpression of PTPN12 (protein tyrosine phosphatase nonreceptor type 12) in NPC cells has decreased EGFR expression. It has enhanced caspase3 activity, preventing the proliferation and invasion of tumor cells [80]. Overall, the EGFR and EGFR signalling pathways play an essential role in the invasion and metastasis of NPC.

Recently, research showed that highly metastatic NPC cells secrete EGFR-rich extracellular vesicles (EVs) that can be absorbed by poorly metastatic NPC cells. Then, EGFR-rich EVs promote EGFR up-regulation and intracellular ROS reduction through the EGFR/PI3K/Akt pathway, thus aggravating the metastasis and progression of NPC [81]. Undoubtedly, EVs delivering EGFR or EGFR ligands promote angiogenesis, metastasis, and osteoclastogenesis in tumors, modulating the immune system and blocking these EVs’ activities to reduce drug resistance [82,83]. Therefore, the EGFR may be a favorable indicator for the progression of NPC, which would be beneficial for exploiting new anti-metastatic medicine for advanced NPC therapies. However, it remains to be studied [84].

### 4.4. Other Roles of EGFR in Nasopharyngeal Carcinoma

DLC-1 (liver cancer-1) can inhibit NPC proliferation, metastasis, and deterioration and is a candidate of NPC tumor suppressor gene. Research into the mechanisms behind the opposing roles of DLC-1 in NPC cells confirmed that the ectopic expression of DLC-1 can induce mitochondrial apoptosis. Furthermore, it also inhibits EMT and other related processes through the EGFR/Akt/NF-κB pathway [85]. This undoubtedly lays a foundation for clinical NPC application [86]. Another study suggested that curcumin inhibits the EGFR, STAT3, and growth factor receptor-bound protein 2 (GRB2) via the circRNA–miRNA–mRNA network, thereby enhancing the radio-sensitization of NPC [87]. The clinical application of anti-EGFR drugs has profound significance. However, the functions of the EGFR in NPC need further research.

## 5. EGFR Effects on EBV Infections

### 5.1. EGFR Is Overexpressed in EBV-Infected Cells

EBV infections induce many EGFR downstream pathways; however, current research has demonstrated that EGFR also gives rise to EBV infection. Studies reported that the EGFR level in EBV-positive NPC patients is significantly higher than in EBV-negative patients. In particular, EGFR and STAT3 have been detected in the nasopharyngeal tissues of EBV-positive patients, and they are up-regulated in the nucleus of epithelial cells and inflammatory cells in EBV-positive chronic nasopharyngitis patients [17,88]. In addition, LMP1 can induce STAT3 phosphorylation, EGFR expression and nuclear accumulation, EGFR and STAT3-dependent inducible nitric oxide synthase (iNOS) expression, and subsequent DNA damage. Thus, it is believed that EGFR and STAT3-dependent pathways play a crucial role in EBV-related tumors [88]. In conclusion, the EGFR may promote the neoplastic transformation of EBV-positive cells.

### 5.2. EGFR Enhances the Internalization and Fusion of EBV

Neuropilin 1 (NRP1) is a co-receptor of class III semaphorins and various growth factors such as EGF, VEGR, TGF-β, and FGF, which synergistically increase receptor tyrosine kinase activity [89]. After EBV contacts epithelial cells, NRP1 can directly interact with EBV gB, a conserved glycoprotein required for membrane fusion in herpesviruses, then recruit EGFR and EGF-binding receptors to up-regulate the expression of NRP1. Subsequently, EBV could activate the EGFR/Akt and EGFR/ERK pathways. Consequently, the EGFR enhances EBV infection by promoting it to enter epithelial cells via macropinocytosis and lipid raft-dependent endocytosis. Hence, the EGFR knock-out could partially inhibit EBV infections that provide an opportunity for NPC treatment [10]. Furthermore, pathogen-ErbB ligation and the ErbB receptor signalling pathway can contribute to the cellular entry of microbes. However, intracellular organisms, such as the EBV, may require ErbB signalling cascades for self-propagation [33]. In general, the EGFR is necessary for the internalization and fusion of the EBV in NPC cells and perhaps can enhance its survival in the host.

## 6. The Role of EGFR Targeting in Nasopharyngeal Carcinoma

Radiotherapy is the primary treatment for the early diagnosis of NPC, and palliative chemotherapy is often used for advanced NPC since EGFR overexpression promotes tumorigenesis and progression via downstream signalling pathways, causing poor outcomes in NPC patients. EGFR-targeted drugs could be considered a newly developed anti-tumor drug for NPC. Anti-EGFR therapy has been extensively applied in the treatment of NPC and has achieved a better therapeutic effect in recent years [90]. Current therapies targeting the EGFR in tumors include monoclonal humanized antibodies (mAb) that target the extracellular domain of the receptor (e.g., Cetuximab (CTX), Nimotuzumab (NTZ), and Panitumumab) and selective small-molecule inhibitors that target the tyrosine kinase domain, such as Gefitinib, Erlotinib, and Afatinib, PI3K inhibitors and antisense gene therapy [32,91].

Clinical trials have shown that EGFR-targeted therapy (CTX and NTZ) based on palliative chemotherapy for recurrent or metastatic NPC (R/M NPC) patients significantly increase the chance of progression-free survival (PFS). Moreover, toxic and non-toxic side effects are within a controllable range, suggesting the importance of anti-EGFR therapy in NPC treatment [92]. CTX and Panitumumab are the most widely used, neutralizing monoclonal antibodies, and inhibit receptor activation and downstream signalling. Furthermore, a combination of anti-EGFR antibodies is more effective than single antibodies [93]. Different subtypes of nasopharyngeal carcinoma with various genomic profiling show diverse outcomes for targeted therapy (Table 1) [94].

### 6.1. EGFR Monoclonal Antibody

Studies have shown that palliative chemotherapy combined with an EGFR monoclonal antibody could significantly prolong overall survival (OS) and PFS of R/M NPC patients. Specifically, for those with locally advanced NPC (LA NPC), EGFR-targeted agents combined with induction chemotherapy have significantly higher disease-free survival (DFS) rates and fewer side effects. Meanwhile, shorter-use cycles of CTX and NTZ (meaning lower cost) in this treatment, compared to EGFR-targeted agents combined with concurrent chemoradiotherapy (CCRT), are instructive for the treatment of LA-NPC [97]. Palliative chemotherapy combined with EGFR treatment did not significantly improve OS with early R/M NPC [98]. Nevertheless, XTC-treated patients have a longer PFS than those who are NTZ-treated, and have an acceptable overall toxicity. Specifically, the toxic effects in patients treated with CTX are more common than in patients treated with NTZ [92]. The curative effect of the EGFR monoclonal antibody combined with radiotherapy, chemotherapy, and CCRT is equivalent to induction chemotherapy combined with CCRT. However, the combination with a monoclonal antibody has fewer side effects [110].

#### 6.1.1. Cetuximab for Nasopharyngeal Carcinoma

Cetuximab, an EGFR-targeted drug that has entered clinical trials, plays an anti-tumor role by binding to the extracellular domain of the EGFR. Poly-ICLC is an immune adjuvant often used to activate mature DC cells. Studies have shown that the increase of mature DC cells caused by Poly-ICLC in combination with EGFR-targeted drugs can increase EGFR-targeted CD8+ T cells in NPC cells, thus providing a better prognosis for NPC [95]. LA NPC patients can benefit from CCRT combined with CTX treatment, and the benefit is undeniable in the T4N3 group. However, its toxic side effects are more significant [96]. In R/M NPC patients, the clinical trial demonstrated that Paclitaxel Carboplatin plus Cetuximab (PCE) could achieve a 58.3% ORR rate, with better efficacy and acceptable side effects. It is worth mentioning that patients can receive this treatment on an outpatient basis [99]. The combination of CTX has become one of the first-line treatment options for patients with advanced nasopharyngeal carcinoma.

#### 6.1.2. Nimotuzumab for Nasopharyngeal Carcinoma

Nimotuzumab (NTZ), an EGFR-targeted drug, plays an anti-tumor role mainly by binding to the extracellular domain of EGFR and inhibiting EGF binding [111]. NTZ inhibits proliferation and promotes apoptosis and anti-vascular survival in EGFR overexpressing tumors. It is worth mentioning that the cutaneous and mucosal toxicity of NTZ is significantly lower than other EGFR-targeted agents [112]. Clinical trial results show that NTZ combined with CCRT can significantly improve a patient’s OS. However, the high cost of NTZ limits its clinical benefit [113,114]. For stage II and III NPC, NTZ combined with first-line chemoradiotherapy can significantly improve the prognosis, which is reflected in the OS improvement, DFS, and distant metastasis-free survival rate of patients [100,115]. For stage II-IVb NPC with a high EGFR expression, patients treated with CRT+NTZ/CTX exhibited better distant metastasis-free survival (DMFS) [116]. Nowadays, palliative chemotherapy is a first-line treatment for R/M-NPC patients for whom palliative care is required [100,101,115]. Therefore, NTZ combinations have become one of the first-line treatment options for advanced NPC patients.

### 6.2. Small Molecule EGFR Tyrosine Kinase Inhibitors (TKIs)

TKIs inhibit EGFR activation chiefly by binding to the tyrosine domain of the EGFR. Among the small-molecule inhibitors of the tyrosine kinase, experiments have shown that Lapatinib can inhibit the invasion and proliferation of nasopharyngeal carcinoma cells and promote their apoptosis [117].

In addition to inhibiting the kinase, Gefitinib also increases the formation of the inactive EGFR dimer, suggesting that Gefitinib could induce faster endocytosis and dimer degradation. Gefitinib has been shown to preferentially target cancer stem-like cells (CSC) and eliminate tumor regrowth in vivo and in vitro for NPC patients with CSC [91,102].

The mechanism of Erlotinib is to compete with adenosine triphosphate to bind the intracellular catalytic domain of EGFR and thereby inhibit EGFR phosphorylation [107]. CNE2 cells treated with Erlotinib or Cisplatin inhibit cell viability and migration ability, increase apoptosis, and enhance tumor sensitivity to radiotherapy/chemotherapy, meaning that Erlotinib and Cisplatin weaken the radiotherapy/chemotherapy resistance of tumor cells [105].

Afatinib inhibits EGFR and ErbB-2 tyrosine kinase activity and suppresses NPC cell proliferation by arresting the cell cycle [108]. The combination of Afatinib and Gemcitabine (GEM) have shown significant anti-tumor efficacy in NPC xenograft models [108,109]. In addition, Erlotinib and Afatinib could enhance the sensitivity of tumors to chemoradiotherapy by inhibiting DNA damage repair [105,108]. However, no clinical trials have shown that TKIs can significantly improve the prognosis of NPC patients [107,118]. Therefore, the use of TKIs is limited to NPC patients.

## 7. Conclusions

The EBV is a type 4 γ herpes virus, consisting of lipoprotein capsules and 162 shell particles [2,15]. Incidence of EBV-positive NPC have grown in recent decades, with worse outcomes than for their EBV-negative counterparts. The malignant degree of nasopharyngeal carcinoma cells increased in the presence of the EBV in both in vivo and in vitro experiments [17]. Now, it is being used as a clinical marker in the clinical diagnosis of NPC [18]. LMP1 is a membrane protein encoded by the EBV, consisting of a short amino acid N-terminal, six hydrophobic alpha-helical transmembrane regions, and a large 200-amino acid cytoplasmic C-terminal tail [19]. 

The EGFR is necessary for cell development and homeostasis, the overexpression of which is common in many malignant tumors, and is a driver of tumorigenesis in various cancers, including NPC [35]. As mentioned above, the EGFR forms homodimers or heterodimers by binding to the ligands, phosphorylating and activating the tyrosine kinase domain in the cytoplasm. Afterwards, the EGFR activates downstream signal transduction, such as the PI3K/Akt/mTOR, JAK/STAT, and Ras/Raf/MAPK pathways. Ultimately, these EGFR pathways affect cell survival, proliferation, oncogene transcription, and other cancer-associated reactions.

Subsequent studies found that the CTAR1 domain at the carboxyl end of LMP1 can drive EGFR up-regulation, while the CTAR2 domain cannot [46,58]. In-depth research found that EGFR levels of CTAR1-solely-expressed C33A cells are remarkably higher than LMP1-expressed C33A cells, indicating that the presence of CTAR2 may impede the ability of CTAR1 [46]. Specifically, LMP1 up-regulates EGFR expression and promotes the nuclear translocation of the EGFR through the NF-κB pathway and the activation of STAT3. On the one hand, LMP1 activates the NF-κB pathway to form p50 dimers, activating Bcl-3, and then forms the p50/Bcl-3 complex, which binds to the EGFR promoter region to up-regulate EGFR expression [43,60]. On the other hand, LMP1–CTAR1 can further upregulate the EGFR after STAT3 activation. This occurs when CTAR1 promotes the tyrosine phosphorylation and activation of STAT3, which further induces the expression of Bcl-3, which then induces the nuclear translocation of Bcl-3 and p50. Bcl-3 binds with p50 as a transcriptionally active complex to activate EGFR expression [46,51]. Nevertheless, it is unknown whether EBV can regulate the EGFR through other encoded proteins.

The EGFR is overexpressed in NPC. Several studies confirmed that its expression is strongly related to NPC cell proliferation, invasion, metastasis, and pathogenesis. The latest research showed that the EGFR enhances the internalization and membrane fusion of the EBV in NPC cells. However, the findings of EGFR-assisted EBV infection are rarely known [10]. Moreover, the entry and invasion of pathogens depend on ErbB family receptors. Thus, other receptors in the ErbB family may drive EBV infection and pathogen-induced cellular transformation [33]. Further study on the relationship between ErbB receptors and EBV infections is needed.

The overexpression of the EGFR is common in NPC. Meanwhile, it promotes tumorigenesis and progression via the EGFR signalling pathway, thus reducing NPC patient survival rates. EGFR-targeting drugs could be considered as NPC anti-tumor drugs, and recently, therapies targeting the EGFR in NPC have included monoclonal humanized antibodies (CTX, NTZ and Panitumumab), selective small molecule inhibitors (Gefitinib, Erlotinib, and Afatinib), PI3K inhibitors, and antisense gene therapy [32,91]. Many clinical studies have shown that monoclonal antibodies against the EGFR can significantly prolong the OS and PFS of middle and advanced NPC patients. However, no clinical studies have shown that selective TKIs can improve the prognosis of patients with middle and advanced NPC, which may require further investigation or clinical trials.

## Figures and Tables

**Table 1:** EGFR-targeted drugs in nasopharyngeal carcinoma.

The Category of Drugs	Target	Therapeutic/Experimental Regimen	The Type of the Test	Results	References
Anti-EGFR antibody	The extracellular domain of the receptor	Palliative chemotherapy plus an anti-EGFR agent (NTZ or CTX) in R/M NPC	Clinical trial	Prolong R/M patients’ OS, PFS	[92]
		CTX plus Poly-ICLC treatment	In vitro experiment	Poly-ICLC could enhance both CTX-mediated innate and adaptive anti-tumor immunity against NPC	[95]
		CCRT plus CTX in LA NPC	Clinical trial	Prolong LA NPC patients’ OS, PFS	[96]
		IC+CTX/NTZ or CCRT + CTX/NTZ in LA NPC patients	Clinical trial	Prolong LA NPC patients’ PFS	[97]
		PCT+CTX/NTZ in de novo metastatic NPC patients.	The clinical trial	Cannot improve de novo metastatic NPC patients‘ prognosis	[98]
		paclitaxel + carboplatin + CTX (PCE) therapy for R/M NPC	Clinical trail	Potentially effective for R/M NPC	[99]
		IMRT+cisplatin+NTZ therapy for LA NPC	Clinical trial	Prolong LA NPC patients’ OS	[100]
		NTZ+ chemotherapy for R/M NPC	Clinical trail	Prolong R/M NPC patients’ OS	[101]
Small molecule EGFR tyrosine kinase inhibitors (TKI)	Tyrosine kinase domain	Gefitinib	In vitro and in vivo experiment	Suppress cancer stem-like cells of NPC xenografts.	[102]
		Gefitinib	In vitro and in vivo experiment	Inhibit two NPC cell lines proliferation	[103]
		FA-GEF-Y90-LPNP	In vitro and in vivo experiment	Exhibit the best in vivo tumor inhibition ability without more systemic toxicity	[104]
		Erlotinib plus radiotherapy/chemotherapy	In vitro experiment	Enhance the sensitivity of tumor cells to radiotherapy/chemotherapy, and weaken radiotherapy/chemotherapy resistance of tumor cells	[105]
		GO-PEG-Erlotinib	In vitro experiment	Suppress NPC cell proliferation, migration, and invasion	[106]
		Erlotinib plus Cisplatin in patients with R/M NPC.	Clinical trial	Maintenance or second-line therapy with Erlotinib after chemotherapy was not effective in RM NPC	[107]
		Afatinib	In vitro experiment	Increase NPC cell radiosensitivity	[108]
		Afatinib or Afatinib combination with gemcitabine	In vitro and in vivo experiment	Single Afatinib induces cell cycle arrest and inhibits the proliferation of NPC cell lines. Afatinib + gemcitabine have an anti-tumor effect in an NPC xenograft model	[109]

NTZ: Nimotuzumab; CTX: Cetuximab; CCRT: concurrent radiochemotherapy; PCT: palliative chemotherapy; R/M NPC: recurrent metastatic nasopharyngeal carcinoma; LA NPC: locally advanced nasopharyngeal carcinoma; OS: overall survival; PFS: progression-free survival; FA-GEF-Y90-LPNP: folic acid (FA) modified, gefitinib (GEF) and yttrium 90 (Y90) co-loaded, core-shell structured lipid–polymer hybrid nanoparticles; GO-PEG-Erlotinib: polyethylene glycol-coated graphene oxide loaded with Erlotinib.
